# Supplementary material for: Scaling between cell cycle duration and wing growth is regulated by Fat-Dachsous signaling in Drosophila
Source: eLife. 2024 Jun 6;12:RP91572. doi: 10.7554/eLife.91572 (PMC11156469; doi:10.7554/eLife.91572)
Supplement: Figure 6—source data 1. [file elife-91572-fig6-data1.docx]

Figure 7 – source data 1

|  | Time_Cell Cycle_=  β_0_ + β_1_ x Volume | |  |  | different from control | |
| --- | --- | --- | --- | --- | --- | --- |
| Genotype | β_0_ | β_1_ | *R^2^* | *p* | *p* of intercept | *p* of slope |
| *nub-Gal4* | 9.480 ± 1.011 | 15.231 ± 1.913 | 0.74 | 6.38E-08 |  |  |
| *nub>fat-HA* | 9.798 ± 1.779 | 16.425 ± 2.833 | 0.57 | 4.81E-06 | 0.875 | 0.726 |
|  | | | | | | |
| *ds-Gal4* | -6.445 ± 4.137 | 23.251 ± 3.771 | 0.864 | 8.35E-04 |  |  |
| *ds>Ds* | 0.025 ± 5.245 | 12.956 ± 4.294 | 0.5 | 1.46E-02 | 0.365 | 0.108 |
