# Supplementary material for: Scaling between cell cycle duration and wing growth is regulated by Fat-Dachsous signaling in Drosophila
Source: eLife. 2024 Jun 6;12:RP91572. doi: 10.7554/eLife.91572 (PMC11156469; doi:10.7554/eLife.91572)
Supplement: Figure 6—source data 2. [file elife-91572-fig6-data2.docx]

Figure 6 – source data 2

|  | Time_Cell Cycle_=  β_0_ + β_1_ x Time | |  |  |
| --- | --- | --- | --- | --- |
| Genotype | β_0_ | β_1_ | *R^2^* | *p* |
| *nub-Gal4* | -11.983 ± 5.238 | 0.283 ± 0.0519 | 0.574 | 1.80E-05 |
| *nub>ds*(RNAi) | -9.766 ± 4.636 | 0.225 ± 0.0453 | 0.529 | 5.66E-05 |
|  | | | | |
| *nub-Gal4* | -30.104 ± 6.727 | 0.390 ± 0.0624 | 0.697 | 8.71E-06 |
| *nub>GFP* (RNAi) | -15.750 ± 3.500 | 0.232 ± 0.0326 | 0.689 | 2.87E-07 |
